# Iron metabolism and its association with dyslipidemia risk in children and adolescents: a cross-sectional study

**DOI:** 10.1186/s12944-019-0985-8

**Published:** 2019-02-12

**Authors:** Yanna Zhu, Baoting He, Yunjun Xiao, Yajun Chen

**Affiliations:** 1 Department of Maternal and Child Health, School of Public Health, and Global Health Institute (SGHI), Sun Yat-sen University, No.74 Zhongshan Road II, Guangzhou, 510080 Guangdong Province China; 2 Department of Nutrition and Food Hygiene, Shenzhen Center for Disease Control and Prevention, Shenzhen, China

**Keywords:** Transferrin, Soluble transferrin receptor, BMI, Dyslipidaemia, Children

## Abstract

**Background:**

Information on the association between iron metabolism and dyslipidaemia in children is limited. Thus, this study aims to evaluate the iron metabolic status of children with different body mass index (BMI) and to examine the association between iron metabolism and dyslipidaemia risk.

**Method:**

In total, 1866 children and adolescents aged 7–18 were enrolled in this study, including 912 boys and 954 girls. In this cross-sectional study, parameters for anthropometry, lipids and iron metabolism including transferrin, soluble transferrin receptor (sTfR), ferritin and serum iron (SF) were evaluated. Data regarding demographic characteristics, diet, and physical activity were collected by self-reported questionnaires.

**Results:**

The prevalence of dyslipidaemia and iron deficiency in children and adolescents increased based on BMI categories (both *P* < 0.05) and were 58.3 and 8.9% in subjects with obesity, respectively. The lowest SF and the highest ferritin levels were observed in subjects who were obese (both *P* < 0.001). Subjects with dyslipidaemia had lower SF, transferrin and sTfR levels by different BMI categories, and those who were obese had higher ferritin levels (all *P* < 0.05). Most importantly, higher concentrations of transferrin and sTfR were related to lower dyslipidaemia risk (*OR* for transferrin: 0.49, 95% *CI*: 0.33–0.71; *OR* for sTfR: 0.68, 95% *CI*: 0.46–0.99).

**Conclusions:**

A downward trend in SF level by BMI categories and the highest ferritin level in subjects with obesity suggested that iron storage was associated with BMI in children and adolescents. Moreover, an inverse relationship was observed between transferrin and sTfR concentrations and dyslipidaemia risk in children with different BMI.

## Introduction

Among children, dyslipidaemia, obesity, and other risk factors have been associated with the initiation and progression of atherosclerosis and subsequent adverse cardiovascular outcomes [1]. In 2011–2012, the prevalence of dyslipidaemia reached 20.2% among children in the US, suggesting that one in five teenagers in the US had adverse lipid concentrations and faced a higher risk of atherosclerosis [2]. The increasing trend in the prevalence of dyslipidaemia among children is likely to continue and could be largely contributing to the prevalence of atherosclerosis in adults [3, 4].

A few decades ago, Sullivan proposed the iron hypothesis, suggesting that iron metabolism contributes to inflammation and oxidative stress, which may lead to atherosclerosis and other cardiovascular diseases [5, 6]. In addition, previous epidemiological studies have reported that iron metabolic alteration, such as ferritin or serum iron, which are known as indicators of iron storage, are associated with oxidative stress and adverse lipid concentration in children and adults [7–9]. Moreover, several studies have reported that transferrin and soluble transferrin receptor (sTfR) are associated with obesity, metabolic syndrome and other cardiovascular risk factors in adults and children [10–12]. However, studies on the relationship between transferrin, sTfR and the risk of dyslipidaemia are still limited, especially in children. Furthermore, a systematic investigation on the association between iron metabolic parameters and the risk of dyslipidaemia in children with different BMI has yet to be performed. The aim of this study was to explore the condition of iron metabolism and lipid profile in children and adolescents with different BMI and examine the association between the risk of dyslipidaemia and iron metabolic parameters.

## Materials and methods

### Study population

This analysis is part of a large epidemiological study on the constitution and health of students which was conducted in the urban area of Guangzhou, South China, in 2014. Five elementary schools and four secondary schools were included in this study based on multistage cluster sampling. Participants were eligible for inclusion in this analysis if the following criteria were met: [1] their age ranged from 7 to 18 years; [2] they did not have genetic syndromes, endocrine diseases, or psychiatric disorders; [3] they were not graduating within 1 year (not in grade 6 or 9). Ultimately, in total, 1866 children and adolescents were recruited. The baseline examination included anthropometric measurements, questionnaires regarding their diet and physical activity and blood sampling.

Approval was obtained from the Ethics Committee of Peking University. Written informed consent was obtained from the participants.

### Questionnaire assessments

Based on previously tested and validated questions, self-reported or parent-reported questionnaires were designed to elicit information on participants’ demographic characteristics, diets, and physical activities. Information regarding diets was collected by calculating food consumption during the last week before the survey, while information regarding physical activities was assessed as vigorous and moderate intensity, which were both calculated as the average time spent per week. The detailed assessment of diet and physical activities can be found in our previous study [13], which was just summarized that for dietary information, similar questions regarding consumption of fruit, vegetable and meat were asked (One serving of the fruit, vegetable and meat was approximated equal to the palm size of an adult). For physical activities, moderate-intensity activities (those having 3–6 Metabolic Equivalents) and vigorous-intensity activities(those having > 6 Metabolic Equivalents) was estimated by giving examples.

### Anthropometric measurements

All participants in this study were subjected to anthropometric measurements. Height, hip and waist circumferences were measured to the nearest 0.1 cm, and weight was measured to the nearest 0.1 kg in light clothing. Body mass index (BMI) was calculated as weight (kg) / [height (m)] [2], and waist-to-hip ratio (WHR) was calculated as waist circumstance (cm) / hip circumstance (cm). Blood pressure including systolic and diastolic blood pressure was measured in the seated position using a mercury sphygmomanometer (Yutu XJ1ID, Shanghai, PRC) with at least a 10-min rest period before the measurement. BMI was categorised into four groups (including thinness, normal weight, overweight and obesity) according to the guideline of the Working Group on Obesity in China.

### Clinical and biochemical measurements

Venous blood samples were collected at baseline from participants on the morning after an overnight fast. After centrifuging, plasma and serum were frozen at − 80 °C until they were analysed for the following: [1] lipid profile including triglyceride (TG), high-density lipoprotein (HDL), high-density lipoprotein (LDL) and total cholesterol (TC) levels, which were measured using routine laboratory methods performed by specialized laboratories accredited by Peking University; [2] Serum iron levels determined by the ferrozine method (SI 7967, Randox Laboratories Co. Ltd., Crumlin, UK); [3] Serum ferritin levels tested by an immunoturbidimetric assay (Fer0060, Jingyuan medical instrument Co. Ltd., Shanghai, PRC); [4] Serum transferrin levels assessed by an immunoturbidimetric assay (Transferrin Reagent No.67758, Orion Diagnostica Oy Co. Ltd., Espoo, Finland); [5] sTfR levels analysed by an immunoturbidimetric assay (12,148,315, Roche Diagnostics Co. Ltd., Indianapolis, US).

### Definitions of iron deficiency and dyslipidaemia

Iron deficiency was defined as serum iron concentrations < 45 μg/dL [14]. Dyslipidaemia in children and adolescents in this study was determined according to the high cut-off values of the National Heart, Lung, and Blood Institute of the US National Institutes of Health [1]. In detail, adolescents were classified as having dyslipidaemia if they met one or more of the following criteria regarding abnormal lipid level: [1] TC ≥200 mg/dL (5.17 mmol/L); [2] LDL ≥130 mg/dL (3.36 mmol/L); [3] TG ≥100 mg/dL for subjects aged 7–9 years (1.13 mmol/L), ≥130 mg/dL for subjects aged 10–18 years (1.47 mmol/L); [4] HDL ≤40 mg/dL (1.03 mmol/L).

### Statistical analysis

EpiData 3.0 software (The EpiData Association, Odense, Denmark) was used to establish the data bank. Normally distributed continuous variables in this study were displayed as the mean and SD, while continuous variables with skewed distributions were displayed as the median with lower and upper quartiles. To normalize the distributions, skewed variables were log-transformed for further statistical analyses. To compare the normally distributed continuous variables, a one-way analysis of variance was performed. To compare the non-normally distributed continuous variables, the Kruskal-Wallis *H* test was used. A post hoc least significant difference (LSD) *t*-test was applied to examine intergroup differences. The odds ratios of dyslipidaemia in different quartiles of each iron parameter were calculated using a binary logistic regression in both the crude model and adjusted model controlling for age, gender, BMI, diet and physical activity. Odds ratios (*ORs*) and 95% confidence intervals (*CIs*) were obtained as measures of association and precision. All *P* values were two-sided, and *P* < 0.05 was considered statistically significant. All analyses were performed using Statistical Package for the Social Science (SPSS) (version 19.0, SPSS Inc. Chicago, IL, USA).

## Results

### Iron parameters and lipid profiles of children and adolescents with different BMI

The characteristics of the subjects included in the study, stratified by their BMI categories, are presented in Table 1. The ages of the subjects ranged from 7 to 18 years old. In total, 912 participants (48.9%) were male. Increasing trends according to BMI categories were observed in the prevalence rates of both dyslipidaemia and iron deficiency. Specifically, significantly higher prevalence rates of both dyslipidaemia and iron deficiency were found in children and adolescents who were obese (58.3 and 8.9%, respectively) and overweight (47.5 and 9.2%, respectively). Among these children and adolescents, the concentrations of TC, TG and LDL-C increased in those who were obese compared to those who were normal weight, whereas the levels of HDL-C decreased, suggesting great differences in the lipid profiles of children and adolescents with different BMI categories (*P*_*trend*_ < 0.001). In addition, serum iron levels trended lower in subjects who were overweight and obese (15.1 μmol/L (*IQR* 11.1–18.7) and 14.9 μmol/L (*IQR* 11.7–18.3), respectively, both *P* < 0.001), and ferritin levels were higher in subjects who were obese (65.0 μg/L (*IQR* 47.0–87.5)) than those who were normal weight (*P* < 0.001). However, no significant differences were observed in transferrin and sTfR levels among children with different BMI categories. Additionally, blood pressure was higher in those who were overweight or obese than those who were normal weight or thinness, whereas no significant differences were found in their diet and duration of physical activity.

**Table 1:** Baseline characteristics of the study population (*n* = 1866) stratified by BMI categories [1]

	Thinness (*n* = 234)	Normal weight (*n* = 1124)	Overweight (*n* = 261)	Obesity (*n* = 247)	*P* for trend
Anthropometry					
Male (n, %)	108 (46.2)	473 (42.1)	154 (59.0)^**^	177 (71.7)^**^	< 0.001
Height (cm)	139.39 ± 15.74^**^	145.73 ± 16.44	148.74 ± 15.43^*^	147.34 ± 14.50	< 0.001
Weight (kg)	27.78 ± 8.91^**^	37.89 ± 12.82	48.90 ± 15.37^**^	55.84 ± 17.94^**^	< 0.001
BMI (kg/m^2^)	13.89 ± 1.23^**^	17.23 ± 2.35	21.42 ± 2.51^**^	25.07 ± 4.62^**^	< 0.001
Biochemistry					
TC (mmol/L)	4.29 ± 0.82	4.23 ± 0.89	4.33 ± 0.76	4.46 ± 0.77^**^	0.002
TG (mmol/L)	0.83 ± 0.40	0.89 ± 0.45	1.08 ± 0.70^**^	1.18 ± 0.57^**^	< 0.001
HDL-C (mmol/L)	1.41 ± 0.35	1.37 ± 0.36	1.26 ± 0.33^**^	1.22 ± 0.29^**^	< 0.001
LDL-C (mmol/L)	2.38 ± 0.68	2.35 ± 0.71	2.46 ± 0.68	2.56 ± 0.67^**^	< 0.001
Serum iron (μmol/L)	16.45 (12.20–20.30)	16.30 (12.60–20.20)	15.10 (11.10–18.70)^**^	14.90 (11.65–18.30)^**^	0.014
Transferrin (g/L)	2.30 (1.97–2.54)	2.32 (2.03–2.63)	2.31 (2.04–2.65)	2.31 (2.04–2.65)	0.566
Ferritin (μg/L)	61.50 (39.00–86.00)	59.00 (41.00–80.00)	55.50 (39.00–77.50)	65.00 (47.00–87.50)^**^	0.072
sTfR (mg/L)	3.10 (2.70–3.80)	3.10 (2.60–3.60)	3.30 (2.70–3.80)	3.10 (2.60–3.85)	0.446
Dyslipidemia (n, %)	83 (35.5)	443 (39.4)	124 (47.5)^*^	144 (58.3)^**^	< 0.001
Iron deficiency (n, %)	13 (5.6)	53 (4.7)	24 (9.2)^**^	22 (8.9)^**^	0.002
Diet					
Fruit (dose/week)	6.00 (4.00–12.00)	7.00 (4.00–12.00)	7.00 (5.00–14.00)	7.00 (5.00–12.00)	0.336
Vegetable (dose/week)	10.00 (7.00–14.00)	14.00 (7.00–14.00)	14.00 (7.00–21.00)^**^	14.00 (7.00–16.25)	0.151
Meat (dose/week)	7.00 (7.00–14.00)	7.00 (7.00–14.00)	7.00 (6.00–14.00)	7.00 (5.00–14.00)	0.592
Sugary beverages (ml/week)	250 (0.00–500.00)	250 (0.00–750.00)	500 (0.00–1000.00)^*^	250 (0.00–1000.00)	0.074
Physical activities					
Vigorous intensity (h/week)	1.58 (0.17–3.62)	1.50 (0.50–4.00)	2.00 (0.67–4.67)^**^	2.00 (0.67–4.83)	0.042
Moderate intensity (h/week)	1.67 (0.50–3.50)	1.67 (0.50–3.50)	2.00 (0.83–4.00)	2.00 (0.58–4.83)	0.009

Abbreviations**:** *BMI* Body mass index, *TC* Total cholesterol, *TG* Triglyceride, *HDL-C* High-density lipoprotein, *LDL-C* Low-density lipoprotein, *sTfR* Soluble transferrin receptor

^1^BMI categories include thinness, normal weight, overweight and obesity

^*^ *P* < 0.05 vs. normal weight; ^**^ *P* < 0.01 vs. normal weight

### Means comparison of iron parameters between children and adolescents with and without dyslipidaemia

**Fig. 1:**
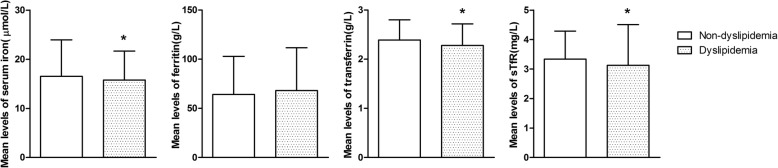
Mean concentrations of serum iron, ferritin, transferrin and sTfR in subjects with or without dyslipidemia. Abbreviation: sTfR, soluble transferrin receptor. * *P* < 0.05 vs. non-dyslipidemia

The levels of iron parameters were further investigated in children and adolescents with and without dyslipidaemia. Figure 1 shows that subjects with dyslipidaemia had average levels of serum iron, transferrin and sTfR that were significantly lower than those without dyslipidaemia (*P* < 0.05), whereas no significant differences were revealed in the ferritin levels between subjects with and without dyslipidaemia. However, significantly lower ferritin levels were observed in obese children and adolescents with dyslipidaemia than those without dyslipidaemia (*P* < 0.05).

### Distribution of iron parameters in children and adolescents with different BMI and lipid categories

**Fig. 2:**
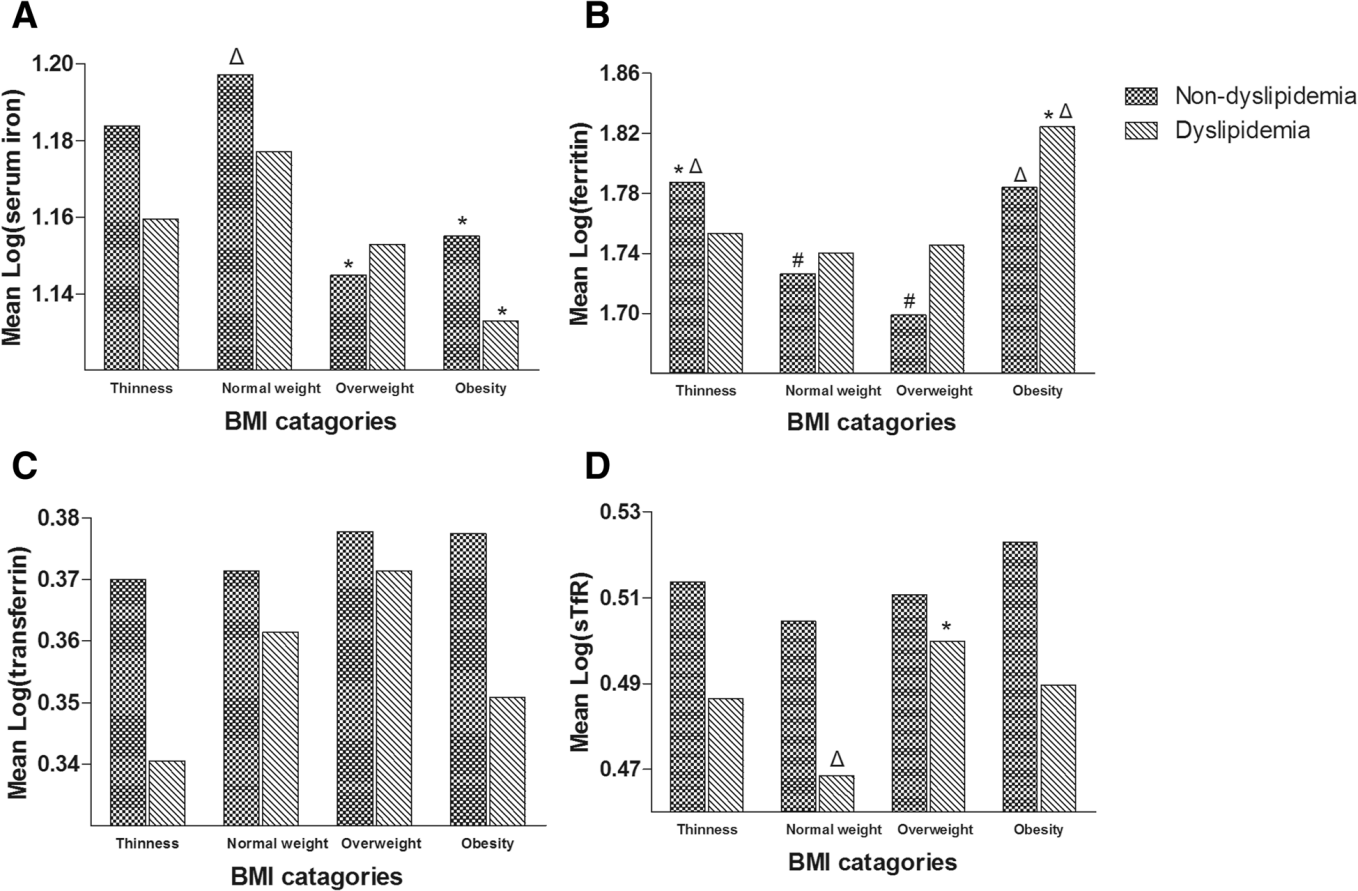
Distribution for the serum iron, ferritin, transferrin and sTfR in subjects with different BMI categories, stratified by dyslipidemia and non-dyslipidemia. Abbreviation: sTfR, soluble transferrin receptor; Log, log-transformed. **a** Serum iron levels after logarithmic conversion; **b** Ferritin levels after logarithmic conversion; **c**. Transferrin levels after after logarithmic conversion; **d** sTfR levels after logarithmic conversion; # Statistically different compared with thinness (*P* < 0.05); * Statistically different compared with normal weight (*P* < 0.05).Δ Statistically different compared with overweight (*P* < 0.05)

Figure 2 shows the distributions of average iron parameter levels after a logarithmic conversion, including serum iron, ferritin, transferrin, and sTfR, in children and adolescents with different BMI categories and lipid status (with or without dyslipidaemia). For subjects with different lipid status, serum iron concentrations did not show a significant difference in non-overweight individuals but exhibited a sharp decrease in those who were overweight and obese. A decrease was shown in the ferritin levels of children with dyslipidaemia who were normal weight and overweight, whereas no significant differences were seen in non-overweight children without dyslipidaemia. In addition, higher ferritin concentrations in children with obesity than overweight children were shown in those with and without dyslipidaemia. The levels of sTfR were higher in overweight subjects than their healthy-weight peers with dyslipidaemia. However, no significant differences or trends were seen in the transferrin concentrations of both groups of subjects with or without dyslipidaemia, and no significant differences or trends were revealed in the sTfR levels of subjects without dyslipidaemia.

### The association between iron metabolism and the risk of dyslipidaemia

Table 2 showed a sub-group analysis in the complete cases with data of both lipid and iron metabolism information. This table indicates that the risk of dyslipidaemia significantly decreased over the quartiles of transferrin and sTfR concentrations, suggesting that the risk of dyslipidaemia was inversely correlated with both transferrin and sTfR concentrations. Compared to subjects in the lowest quartile, subjects in the highest quartiles of transferrin concentrations had a 51% lower risk of dyslipidaemia (*OR*: 0.49, 95% *CI*: 0.33–0.71, *P* < 0.001), while subjects in the highest quartiles of sTfR concentrations had a 32% lower risk of dyslipidaemia (*OR*: 0.68, 95% *CI*: 0.46–0.99, *P* < 0.001) after adjusting for age, gender, BMI, diet and physical activity. However, no significant associations were observed between participants’ risk of dyslipidaemia and their serum iron and ferritin concentrations.

**Table 2:** Risk of dyslipidemia segregated by quartiles of hepcidin, serum iron, ferritin, transferrin and sTfR

	Q1	Q2	Q3	Q4	*P* for trend
Serum Iron					
Male(μmol/L)	< 12.30	12.30–15.70	15.70–20.00	> 20.00	
Female (μmol/L)	< 12.10	12.10–15.90	15.90–19.40	> 19.40	
Dyslipidemia (n, %)	184 (46.7)	187 (44.5)	163 (41.4)	172 (42.9)	
Crude OR	1	0.92 (0.70,1.21)	0.81 (0.61,1.07)	0.86 (0.65,1.13)	0.192
Adjusted OR^1^	1	1.01 (0.70,1.47)	0.89 (0.61,1.29)	0.94 (0.65,1.37)	0.589
Transferrin					
Male(μg/L)	< 2.04	2.04–2.33	2.33–2.65	> 2.65	
Female (μg/L)	< 2.04	2.04–2.31	2.31–2.63	> 2.63	
Dyslipidemia (n, %)	221 (55.3)	184 (44.9)	146 (36.2)	151 (38.2)	
Crude OR	1	**0.66 (0.50,0.87)** ^**^	**0.46 (0.35,0.61)** ^**^	**0.50 (0.38,0.67)** ^**^	< 0.001
Adjusted OR^1^	1	**0.66 (0.45,0.95)** ^*^	**0.51 (0.34,0.74)** ^**^	**0.49 (0.33,0.71)** ^**^	0.015
Ferritin					
Male(g/L)	< 46.00	46.00–64.00	64.00–90.00	> 90.00	
Female(g/L)	< 37.00	37.00–55.00	55.00–74.00	> 74.00	
Dyslipidemia (n, %)	163 (43.0)	185 (44.0)	162 (42.9)	180 (46.2)	
Crude OR	1	1.04 (0.78,1.38)	0.99 (0.75,1.33)	1.19 (0.89,1.58)	0.312
Adjusted OR^1^	1	1.31 (0.90,1.91)	1.19 (0.81,1.75)	1.15 (0.78,1.70)	0.644
sTfR					
Male(mg/L)	< 2.70	2.70–3.20	3.20–3.80	> 3.80	
Female(mg/L)	< 2.60	2.60–3.10	3.10–3.70	> 3.70	
Dyslipidemia (n, %)	206 (55.6)	203 (44.1)	149 (35.9)	144 (39.8)	
Crude OR	1	**0.63 (0.48,0.83)** ^**^	**0.45 (0.34,0.60)** ^**^	**0.53 (0.39,0.71)** ^**^	< 0.001
Adjusted OR ^1^	1	**0.62 (0.43,0.90)** ^*^	**0.54 (0.38,0.79)** ^**^	**0.68 (0.46,0.99)** ^*^	0.026

Abbreviation: *sTfR* Soluble transferrin receptor;

^1^Adjusted for age, gender, BMI, diet and physical activities

^*^P < 0.05;

^**^P < 0.01

## Discussion

Several studies previously revealed that iron metabolic disorder was strongly related to atherosclerosis and other adverse cardiovascular events [15, 16]. However, the association between iron metabolism and the risk of dyslipidaemia has been less systematically studied in children. Thus, the present study examined the condition of iron metabolism and investigated its association with the risk of dyslipidaemia among children and adolescents with different BMI categories. We found that serum iron levels decreased with children’s BMI from normal weight to obesity and reached the minimum level in those with obesity, whereas ferritin levels reached the maximum level in those with obesity. Furthermore, the concentrations of transferrin and sTfR in subjects with obesity or overweight did not differ significantly from non-obese children. However, children and adolescents with dyslipidaemia had lower levels of both transferrin and sTfR concentrations compared to those without dyslipidaemia. Most importantly, the levels of transferrin and sTfR were inversely related to the risk of dyslipidaemia in children and adolescents with different BMI.

### Serum iron and its association with BMI categories and dyslipidaemia

In the present study, the prevalence of iron deficiency and dyslipidaemia in children and adolescents both increased with participants’ BMI categories from normal weight to obesity. Moreover, the prevalence rate of dyslipidaemia in this study was higher than the national prevalence rate because our study was conducted with children living in a high-income urban area of Guangzhou. In addition, an inverse linear trend in BMI categories was also observed in the serum iron levels of all participants in this study. These findings concurred with previous epidemiological studies conducted in both children and adults, suggesting that iron deficiency is more common in overweight or obese subjects than healthy-weight subjects [17, 18]. The current hypothesis connecting obesity and iron deficiency is that obese subjects are exposed to low-grade systematic inflammation, which leads to an increased expression of hepcidin, and hepcidin would diminish iron absorption and down-regulate iron levels in plasma by degrading ferroportin in intestinal enterocytes [19]. Meanwhile, our results also indicated that serum iron levels in children with dyslipidaemia were lower than children without dyslipidaemia. Since children with dyslipidaemia were generally obese and overweight, the mechanism underlying obesity and iron deficiency may have also explained the decreased serum iron levels in those with dyslipidaemia. In addition, according to the iron hypothesis, excessive iron sequestration within macrophages promotes oxidative reactions and the uptake of lipids due to the down-regulation of ferroportin by hepcidin, and it thus facilitates a macrophage accumulation of plaque, which is demonstrated to lead to vessel wall injury and an increased risk of atherosclerosis [20, 21]. Moreover, a study conducted with iron-deficient rats reported that iron deficiency also affected lipid metabolism by down-regulating Apolipoprotein H expression [22], which although not thoroughly studied, has been related to increased cholesterol, TG and LDL in plasma. However, another study conducted in paediatric patients with glycogen storage disease did not show significant differences in serum iron levels between children with and without dyslipidaemia [23]. These differences may be partially explained by the low number of participants in this previous study.

### Ferritin and its trend with BMI categories

Consistent with previous studies [8, 24], higher ferritin levels in children with obesity were observed in the present study. In addition, studies with adults also suggested that ferritin levels were positively associated with BMI [25]. These findings are in line with the observation that obesity is an inflammatory state that increases acute-phase reactants. Ferritin acts not only as a parameter of iron storage but also as an acute-phase protein; therefore, its level in plasma increased in response to obesity-related inflammation [26, 27]. In addition, the ferritin levels in children with obesity were significantly higher in those with dyslipidaemia than those without dyslipidaemia. The underlying mechanism of this finding may have been the different levels of obesity-related inflammatory states between obese subjects with and without dyslipidaemia. Our observation also revealed that ferritin levels declined as BMI categories changed from thinness to overweight in children without dyslipidaemia, whereas it remained stable in those with dyslipidaemia, indicating that the response of ferritin towards inflammation was quite different in subjects with and without dyslipidaemia.

### Status of transferrin and sTfR and its association with dyslipidaemia

Several studies have examined sTfR levels in subjects with metabolic syndrome, but their results are quite inconsistent [11, 28–30]. In addition, a meta-analysis of prospective studies demonstrated a negative relationship between transferrin saturation and cardiovascular events such as coronary heart disease and acute myocardial infarction [31]. These studies mentioned above suggest that there are associations between transferrin and sTfR levels and metabolic syndrome and subsequent cardiovascular events. Still, there is scarce evidence on the relationship between transferrin and sTfR levels and dyslipidaemia, especially in children. In the present study, we observed that levels of transferrin and sTfR in children with dyslipidaemia were lower than subjects without dyslipidaemia, although the concentrations of transferrin and sTfR in this study did not significantly differ by BMI categories. As decreased transferrin and sTfR concentrations indicate reduced bone marrow erythropoietic activity [32, 33], it could be inferred that erythropoiesis is less active in children and adolescents with dyslipidaemia. This may be because increased hepcidin levels may lead to iron sequestration within the reticuloendothelial system and decreased absorption of iron from individuals’ diets, resulting in diminished availability of iron for erythropoiesis.

The present study also revealed that transferrin and sTfR concentrations were inversely associated with the risk of dyslipidaemia in human beings; however, the underlying mechanisms of the relationship are largely unknown. As non-transferrin-bound iron may trigger oxidative damage and an inflammatory response [34, 35], we speculate that increasing transferrin and sTfR concentrations would probably prevent dyslipidaemia and lipid deposition, the early stage of atherosclerosis, by acting as iron-binding proteins and thus reducing labile iron and the subsequent production of very damaging oxidative radicals. Alternatively, it is possible that dyslipidaemia contributes to iron metabolism disorders, resulting in decreased transferrin and sTfR [36]. However, several studies conducted with adults suggested that elevated transferrin concentrations were associated with higher lipids levels [10] likely due to differences between adults and children.

Several limitations of our analysis should be mentioned. First, we defined iron deficiency only by using a serum iron cut-off point, which cannot systematically describe iron deficiency and anaemia. However, we believe that this analysis demonstrates the condition of serum iron and ferritin over different levels of BMI separately and clearly. Second, since the study used a cross-sectional design, the causal sequence underlying the relationships between iron metabolism and dyslipidaemia can barely be detected.

## Conclusion

Overall, iron metabolic parameters were relevant to both the BMI and lipid profile of children and adolescents. On one hand, serum iron concentration trended lower across levels of BMI, whereas ferritin concentrations reached their maximum in subjects with obesity, suggesting that iron storage was closely associated with BMI in children and adolescents. On the other hand, children and adolescents with dyslipidaemia had lower levels of serum iron, transferrin and sTfR, and higher ferritin levels in those who were obese. Furthermore, significant inverse associations were found between the concentrations of transferrin and sTfR and the risk of dyslipidaemia in childhood, which may inversely relate to the risk of atherosclerosis and related cardiovascular disease in adulthood.

## Abbreviations

BMI: Body mass index
CI: Confidence interval
CVDs: Cardiovascular diseases
DBP: Diastolic blood pressure
HDL: High-density lipoprotein
LDL: Low-density lipoprotein
OR: Odds ratio
SBP: Systolic blood pressure
sTfR: Soluble transferrin receptor
TC: Total cholesterol
TG: Triglyceride
WHR: Waist to hip ratio
